# Supplementary figures and images for: Tubulin monoglutamylation is sufficient to rescue the ciliary motility defects in a Chlamydomonas polyglutamylation deficient mutant
Source: Front Cell Dev Biol. 2026 Jun 5;14:1857091. doi: 10.3389/fcell.2026.1857091 (PMC13279043; doi:10.3389/fcell.2026.1857091)

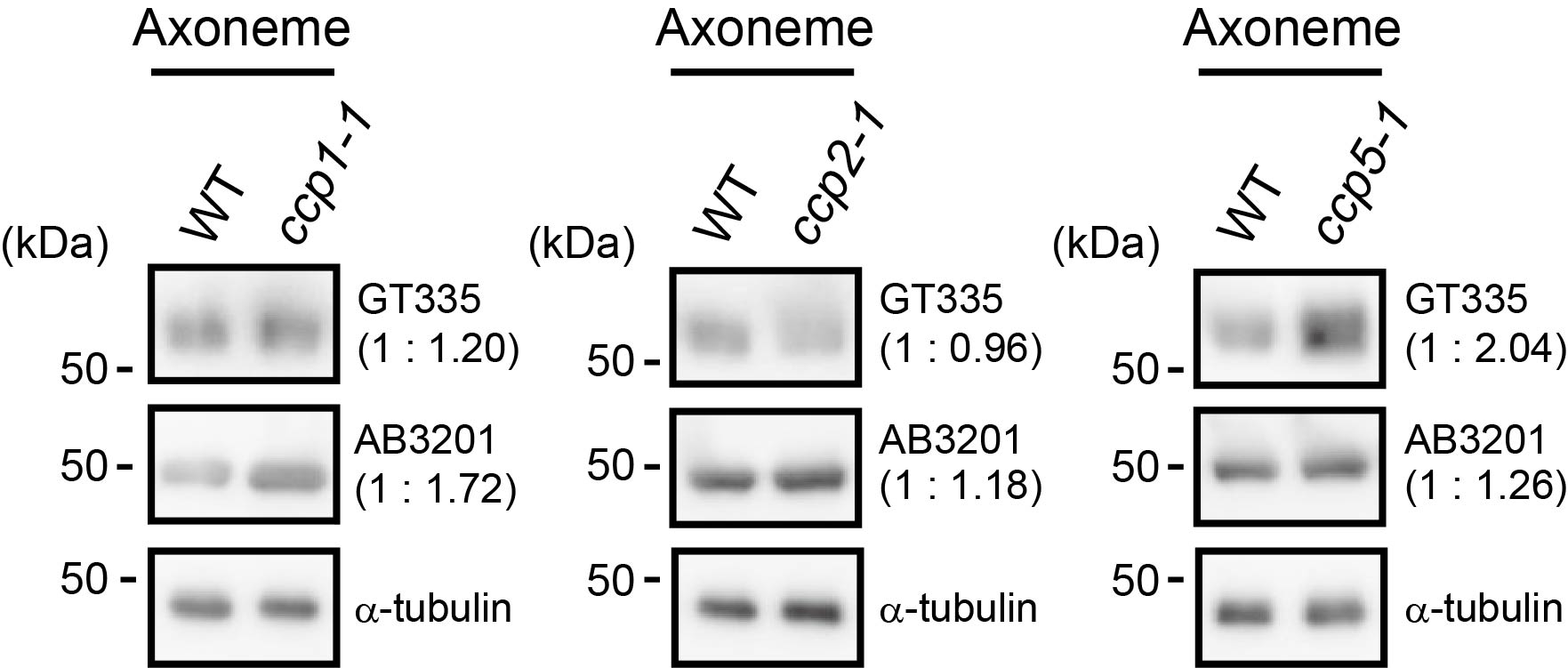

Supplement: Supplementary file 1 [file Image1.jpeg]
